# Temperamental Constellations and School Readiness: A MultiVariate Approach

**DOI:** 10.3390/ijerph18010055

**Published:** 2020-12-23

**Authors:** Andrew S. White, Kate M. Sirota, Scott R. Frohn, Sara E. Swenson, Kathleen Moritz Rudasill

**Affiliations:** 1 Department of Educational Psychology, University of Nebraska–Lincoln, Lincoln, NE 68588, USA; whitedrews@gmail.edu (A.S.W.); katesirota@huskers.unl.edu (K.M.S.); sara.swenson@gmail.com (S.E.S.); 2 PSI Services LLC, Glendale, CA 91203, USA; scottfrohn@gmail.com; 3 School of Education, Virginia Commonwealth University, Richmond, VA 23284, USA

**Keywords:** temperament, effortful control, school readiness, canonical correlation, SECCYD

## Abstract

This study uses canonical correlation analyses to explore the relationship between multiple predictors of school readiness (i.e., academic readiness, social readiness, and teacher-child relationship) and multiple temperamental traits using data from the second wave (age 54 months, *n* = 1226) of the longitudinal Study of Early Child Care and Youth Development (SECCYD; NICHD ECCRN 1993). This longitudinal study collected data on a large cohort of children and their families from birth through age 15. For academic readiness, only one temperamental constellation emerged, representing the construct of effortful control (i.e., high attentional focusing, high inhibitory control). For peer interactions, two significant constellations emerged: “dysregulated” (low inhibitory control, low shyness, and high activity), and “withdrawn” (high shyness, low inhibitory control, low attentional focusing). Finally, the analyses exploring child-teacher relationships revealed two significant constellations: “highly surgent” (high activity, low inhibitory control, low shyness) and “emotionally controlled” (low anger/frustration and high inhibitory control). Results of this study form a more nuanced exploration of relationships between temperamental traits and indicators of school readiness than can be found in the extant literature, and will provide the groundwork for future research to test specific hypotheses related to the effect temperamental constellations have on children’s school readiness.

## 1. Introduction

Children’s successful adjustment to formal school is rooted, in part, in their temperamental traits (e.g., [1,2]), including reactive traits, such as shyness [3], and regulatory traits, such as inhibitory control [4,5]. However, previous studies generally explored specific temperament traits in isolation, often ignoring their interplay [6]. The extent to which a child is academically and socio-emotionally ready for formal schooling (i.e., school readiness) is characterized by multiple indicators [7]; thus, univariate approaches are not ideal for fully capturing the complexity of the relationships between temperament and school readiness. Therefore, in this study, we used multivariate statistical analyses (i.e., canonical correlations) to predict multiple indicators of school readiness using composites of temperamental traits in an attempt to more comprehensively explain this relationship.

### 1.1. School Readiness

School readiness is a broad, multidimensional construct that explains children’s characteristics, present before school entry, that are predictive of positive outcomes in academic settings [8]. School readiness may be conceptualized via academic and social-emotional lenses. Academic readiness comprises emergent academic skills (i.e., literacy, math) that provide necessary foundations for academic achievement (e.g., [9,10,11]). Social-emotional readiness encompasses the social and behavioral competencies that facilitate children’s success in academic settings. It is critical to determine factors related to these dimensions of readiness, as developmental disparities in the early elementary years produce a gap in academic achievement that becomes increasingly difficult to address with the passage of time [12,13]. While research has explored the role of environmental factors related to readiness, such as economic disparities and exposure to opportunities to foster emergent skills [14], less focus has been placed on understanding how the individual characteristics of children guide their interactions with their environments and thus may facilitate or impede school readiness. Therefore, a need exists to further explore the role of children’s temperament in early readiness for school.

### 1.2. Temperamental Dimensions

*Temperament* represents biologically based individual differences in children’s emotional or behavioral reactions to environmental stimuli (e.g., activity level, anger, fear), as well as differences in their ability to regulate those reactions (e.g., effortful control; [15]). Temperament theory aligns with the theoretical framework of *goodness-of-fit* [16], or the extent to which a child’s parent or caregiver consistently provides appropriate developmental support for a child based on their temperamental needs. Good fit enhances children’s psychological development by providing positive support for the growth of regulatory capacities that help children manage their natural reactions in adaptive ways [16]. Thus, while the general blueprint of children’s reactive and regulatory tendencies is present at birth, environmental interactions and experiences will increasingly moderate how temperament is expressed [17].

Temperament theory and research indicate that one’s temperament simply cannot be viewed in isolation [15,18,19,20]. A risk factor for successful transition to kindergarten, such as low effortful control, may be reduced or eliminated by positive social environments in the classroom [21]. Leveraging the powerful relationship between temperament and environment is of prime importance in the earlier school years when children’s traits are especially malleable [21,22].

Children’s temperament is frequently measured using the Children’s Behavior Questionnaire (CBQ; [23], a parent and teacher-report questionnaire of 15 temperament traits, or dimensions, which comprise three factors: Surgency (activity level, impulsivity, high-intensity pleasure, and shyness), negative affect (anger/frustration, discomfort, fear, sadness, and soothability), and effortful control (attentional focusing, inhibitory control, low-intensity pleasure, and perceptual sensitivity). Each factor presents implications for children’s school readiness, which we will briefly outline.

#### 1.2.1. Surgency

*Surgency* comprises dimensions of temperament that align with children’s tendencies to approach or withdraw from given stimuli, and includes activity level, impulsivity, high-intensity pleasure, and shyness. Surgency has been linked to children’s school readiness and classroom outcomes broadly and, specifically, via activity level and shyness. Children high in surgency exhibit a high activity level, positive affect, and approach tendencies, especially when faced with new or potentially rewarding experiences [18,24]. High Surgency is also characterized by low shyness and high sociability [23,25]. The positive affect and reward-seeking approach tendencies of highly surgent children offer robust support for the development of social skills, emotional resilience, and the ability to focus on desired outcomes [26,27,28,29,30,31,32]. 

However, surgency may become a risk factor for children if they cannot regulate their strong approach tendencies [18,33,34,35]. High surgency and impulsivity in combination have been linked to externalizing behavior problems [36,37,38,39]. Children with this temperament combination may quickly become angry or aggressive if a reward is blocked, disregard rules or instructions, or struggle with reflection of their own behavior [18,27,31,40,41,42]. Socially, they may experience more conflict with peers and, in rare cases, be at risk for peer rejection [18,20,43,44]. 

Low surgency, exemplified by the dimension of shyness, also presents significant risk to children’s school readiness. Shy children may be fearful of peer interactions [45,46], and more likely to avoid taking risks and asking for help from teachers or peers [47,48]. Shy children may appear to daydream or disengage from the classroom environment [49,50] reducing their access to critical opportunities for communicative skill practice (e.g., [51,52,53,54,55]). Additionally, withdrawal from social interactions may limit teachers’ ability to identify their shy students’ academic strengths and needs [21,56,57,58,59,60]. Finally, shy elementary children report lower levels of self-esteem than their non-shy classmates (e.g., [46,61,62]. Shy children often struggle with anxiety or helplessness in testing situations [63], and perform worse on standardized tests than non-shy children with similar intelligence [64,65]. Coplan and Rudasill suggest that shy children’s low self-esteem represents the most serious long-term risk to childhood’s most important developmental outcomes, social competence, academic skills, and general mental health and emotional stability [66].

#### 1.2.2. Negative Affect

*Negative affect* (anger/frustration, discomfort, fear, sadness, soothability) refers to temperamental dimensions indicative of children’s negative responses to environmental stressors. For example, some children become easily frustrated when interrupted during a desirable activity (e.g., being asked to put down a toy) or experience more fear in the anticipation of future distress (e.g., announcement of a substitute teacher), whereas other children are able to move on to another activity or distract themselves from a stressful upcoming event. Children with more negative affect tend to be less adaptable; thus, change is stressful and more difficult to manage. Given the stresses of the transition to Kindergarten, children entering the classroom with low adaptability may be at particular risk. Extant research has historically demonstrated a relationship between negative affect and children’s problem behaviors (e.g., [67,68]) implicated in early academic achievement [69]. Preschool children with more intense and negative affective expression are rated by teachers as less teachable [70] and have lower elementary academic achievement [71] than children with more moderate or positive affectivity. 

Longitudinal research has explored the relationships between negative affective traits and surgent traits, such that highly surgent children (high activity, impulsive, rapid approach, and high-intensity pleasure) tend to become more easily frustrated [68,72]. The relationship between approach and negative affect appears to be instead contingent upon the intensity of the situation, such that low-intensity approach is only related to positive emotionality, whereas both positive and negative emotionality relate to high-intensity approach (e.g., [42,73]). Thus, high intensity situations appear to present the greatest challenge to children with high levels of negative affect and highly surgent traits. 

Crucial to consideration of the role of negative affect to school readiness is children’s ability to regulate their predispositions toward negative affect. The relationship between negative affectivity and internalizing and externalizing problems is moderated by children’s capacity to regulate their emotions and behavior [15], such that children with greater negative affectivity experience fewer problems when they are also highly regulated. That is, children with greater regulatory abilities are better prepared to “override” their initial instincts toward emotionality which may interfere with academic or social achievement, leaving children low in regulatory ability particularly vulnerable to the negative outcomes related with intense emotionality [74]. This regulatory construct represents *effortful control*, the final temperamental dimension explored in this paper. 

#### 1.2.3. Effortful Control

In contrast to negative affectivity and surgency, *effortful control* comprises those predispositions that enable children to regulate their natural reactions to the environment [15]. Effortful control is the extent to which a child can either activate or inhibit an inappropriate response and act appropriately in a given situation; it also includes the child’s ability to focus his or her attention, as needed. Research on school readiness underscores the importance of nurturing the development of effortful control for children during the preschool years to prepare for the transition to kindergarten. Children’s effortful control in preschool is positively related to gains in academic skills through the school year [75]. Levels of effortful control at the end of the preschool years predict a child’s reading achievement [76] and general academic adjustment in kindergarten [77]. Children entering kindergarten with higher levels of effortful control also report greater academic gains at the end of the year (e.g., [78]). For children at particular risk at the transition to kindergarten (i.e., low-income students) the development of effortful control in preschool may be even more critical [2,77]. 

Notably, given a classroom environment that supports the improvement of a child’s regulatory skills, effortful control is a trait that can also be developed through practice [79]. However, some research suggests that students make the most gains when there is a match between their level of effortful control (i.e., high or low), and their teacher’s level of this trait (e.g., “goodness of fit) [80]. In contrast, a mismatch between teacher and student regulatory levels can lead to relational strain between student and teacher, especially if the teacher is very highly regulated [80]. These findings support not only the critical importance of effortful control in the classroom, but also highlight the importance of the teacher-child relationship for its growth during the early school years. 

### 1.3. Purpose and Research Questions

Given our interest in the potential impact of all of temperament traits on children’s outcomes, and the potential for school interventions that improve goodness-of-fit, the purpose of this study is to determine how multiple temperamental traits combine to predict children’s school readiness. Extant literature has generally explored specific temperament traits, rather than the interplay among them [6]. Because school readiness is most accurately described as a function of multiple dimensions of traits (i.e., constellations of traits) [7], univariate approaches to analysis are likely unable to fully capture the complexity of the relationships between temperament and school readiness. 

This study therefore builds upon existing literature connecting temperamental traits with school readiness through the use of multivariate statistical analyses to predict multiple indicators of school readiness using multiple temperamental traits. In this study, we addressed three questions. First, how do constellations of temperamental traits predict the academic readiness of incoming kindergarten students? Second, how do constellations of temperamental traits predict the social-emotional readiness of incoming kindergarten students, as indicated by the nature of a students’ peer interactions? Third, how do constellations of temperamental traits predict the social-emotional readiness of incoming kindergarten students, as indicated by teacher-child relationship quality?

## 2. Materials and Methods

### 2.1. Participants

The current study makes use of the National Institute of Child Health and Human Development Study of Early Child Care and Youth Development (SECCYD; [81]) dataset. The original data were gathered from children (*n* = 1364) and their families at birth; these children (and families) were then followed longitudinally through the age of 15 years. Recruited families lived in 10 cities in the United States; children were born in 1991. For this study, data from the second wave of the study (age 54 months to 1st grade; *n =* 1226) were used. Canonical correlation requires listwise deletion for participants without complete data, and those with outliers (based on Malhanobis distances) were also deleted. Thus, we had slightly different sample sizes for each analysis. For temperament and school readiness, *n* = 619 (312 boys, 84% White), for temperament and peer interactions, *n* = 637 (322 boys, 85% White), and for temperament and teacher-child relationship quality, *n* = 655 (324 boys, 84% White). Ninety-six percent of participating kindergarten teachers were female and White. 

A series of ANOVAs were conducted to determine whether the final samples for each set of canonical correlations differed significantly on any of the key variables (e.g., teacher-child closeness) from the participants who were not included in the sample; there were no significant differences. Chi-square analyses were conducted to determine whether the gender and ethnic distributions for the subsamples were significantly different from those who were not included in analyses. There was no difference in gender distributions. In terms of ethnicity, there were significant differences in the distributions for two of the analyses. For the temperament and peer interactions, analysis showed a difference (χ^2^_4_ = 12.54, *p* = 0.014); specifically, there were fewer Black/African American students in the analysis sample (*n* = 64) than the deleted sample (*n* = 112). The temperament and teacher-child relationship analysis showed a difference (χ^2^_4_ = 10.08, *p* = 0.039); there were significantly fewer Black/African American (*n* = 69) and students of other races/ethnicities (*n* = 25) than the deleted sample (*n* = 109 and 39, respectively). 

### 2.2. Measures

#### 2.2.1. Child Temperament

When children were 54 months of age, parents reported on child temperament using a subset of scales from the Children’s Behavior Questionnaire [23], representing dimensions from each of the three factors. For Surgency, the scales included activity level (*α* = 0.71), approach (*α* = 0.65), and shyness (*α* = 0.87). For Negative Affect, the scales included anger/frustration (*α* = 0.78), fear (*α* = 0.64), and sadness (*α* = 0.60). For effortful control, the scales included attentional focusing (*α* = 0.74) and inhibitory control (*α* = 0.74). 

#### 2.2.2. Academic Readiness

Academic measures of school readiness were selected for inclusion in the present study. Measures included standard scores from five subscales of the Woodcock-Johnson Psycho-Educational Battery-Revised, collected when children were 54 months old [82,83]: applied problems (*α* = 0.84), incomplete words (*α* = 0.86), letter-word identification (*α* = 0.84)*,* memory for sentences (*α* = 0.82)*,* and picture vocabulary (*α* = 0.76). We also used scores from two subscales of the Academic Rating Scale (ARS; [84]), a teacher-report measure collected when the children were in kindergarten: language and literacy (*α* = 0.87), and mathematical thinking (*α* = 0.92). 

#### 2.2.3. Social Readiness

Children’s kindergarten teachers reported on multiple aspects of children’s social interactions with peers. Three measures from the Social Skills Rating System (SSRS; [85]) were included: self-control (e.g., “controls temper when arguing with other children”; *α* = 0.87), cooperation (e.g., “makes friends easily”; *α* = 0.92), and assertion (e.g., “makes friends easily”; *α* = 0.86). The SSRS has a three-point rating scales where 0 = *never* and 2 = *very often*. Teachers also rated child peer status (4 items, *α* = 0.80). A sample item is “Are there children who like to play or work with the study child?” (1 = *none*, 5 = *nearly all*). Teachers responded to a single *sociometric status* item on a 7-point scale (“This child is well liked by peers”). Kindergarten teachers rated their relationships with the target child using the short form of the Student Teacher Relationship Scale (STRS; [86]). This measure uses 15 items with a five-point scale (1 = *definitely does not apply* to 5 = *definitely applies*) assessing teacher and student relationships, completed individually for each student, and provides subscales representing teacher-student conflict (α = 0.90) and closeness (α = 0.86). 

### 2.3. Procedures

Children’s temperament data were gathered from parents when children were 54 months old. WJ-R data were collected in researcher labs when children were 54 months old. Teacher-reported data (i.e., ARS, SSRS, peer status, sociometric status, and STRS) were reported by children’s teachers during the spring of kindergarten.

### 2.4. Data Analysis

Given our desire to examine both the complex relationships between multivariate constructs (child temperament, school readiness), and the combined effects of certain sets of variables on school readiness outcomes, we conducted canonical correlation analysis (CCA). CCA is a correlational multivariate technique in the General Linear Model that estimates two synthetic variables—each comprised of components of factors from a variable set—that are maximally correlated with one another. It is an optimal analytical approach for examining statistical relationships between two sets of variables because it allows for multiple, simultaneous tests of associations [87]. These canonical functions are called *variates*, and a function relating to one of the variable sets is comparable to a set of standardized weights in multiple regression (referred to in CCA as *standardized canonical function coefficients*). The correlation between the two synthetic variables is referred to as a *canonical correlation coefficient,* equivalent to a Pearson *r*, with the squared canonical correlation representing the shared proportion of variance between the variables. After one canonical variate is estimated, another orthogonal function is estimated with the residual variance from the initial variate. The number of possible canonical variates is equal to the fewest number of indicators in either variable set; yet, as a general standard, only variates that explain a considerable portion of the shared variance (i.e., 10–20% or more) are subjected to further interpretation [87]. An important assumption of CCA is multivariate normality [87,88].

Among the data in the present study, four items demonstrated substantial negative skew (SSRS self-control and cooperation, peer status, and teacher-child closeness). These variables were rescaled so that the minimum value was equal to 1.0, then transformed by squaring the rescaled variable. One item demonstrated substantial positive skew (teacher-child conflict). This variable was rescaled in the same manner, then transformed by taking the natural log of the rescaled variable. Using a graphical assessment of a χ^2^ versus Mahalanobis distance plot, multivariate normality was evaluated for each pair of variable sets [89] as follows: temperament and (1) academic readiness, (2) peer interactions, and (3) teacher-child relationship. Outlier cases causing violation of multivariate normality were identified for each pair of variable sets and omitted from subsequent analyses (*n* = 8 for temperament and school readiness; *n* = 5 for temperament and peer interactions; *n* = 4 for temperament and teacher-child relationship).

CCA was conducted for each variable set pair using SPSS. First, full model fit was assessed using Wilks’s lambda, which indicates whether the full model is statistically significant and provides a measure of effect size (1 − λ = full model *R*^2^). Second, canonical variates were then evaluated for meaningfulness, with only those explaining a significant portion of variance (i.e., >10%) between the sets interpreted (see [90,91] for a discussion of interpreting meaningfulness of canonical variates). Third, functions were hierarchically tested for significance via dimension reduction analysis. Fourth, standardized canonical function coefficients and structure coefficients were evaluated. Function coefficients are weights applied to observed variables used to produce standardized synthetic scores (similar to β weights in regression). Structure coefficients refer to the correlation between observed variables and the synthetic variables, and are usually preferred over function coefficients for interpretational use when intercorrelation is likely between variables in a set [91]. However, high canonical coefficients in relation to other variables in a set may also be considered as meaningful for interpretation of the data [91].

## 3. Results

### 3.1. To What Extent Do Constellations of Temperamental Traits Predict the Academic Readiness of Incoming Kindergarten Students?

Canonical correlation analysis of eight temperament dimensions predicting seven school readiness variables yielded seven canonical correlations (or variates). The full model including all functions was significant (Wilks’s λ = 0.772; *F* [56, 3214.87] = 2.83, *p* < 0.001), where the full model effect size, similar to an *r*^2^, was equal to (1 − λ), or 0.228. This can be interpreted as the full model explaining 22.8% of the variance between the temperament variable set and the school readiness variable set. Of these seven, only the first canonical variate was statistically significant; subsequent variates were not significant at *p* < 0.05. Additionally, the first variate explained a considerable portion of shared variance between variable sets ($R_{s}^{2}$ = 0.171); subsequent functions (2–7) explained very small and non-significant portions of shared variance ($R_{s}^{2}$ = 0.044 to $R_{s}^{2}$ = 0.001). 

Standardized canonical function coefficients and structure coefficients for the first variate of the temperament and school readiness variable sets are presented in Table 1. Examining the coefficients, the predictor variables of attentional focusing and inhibitory control comprised the majority of the synthetic predictor variable, whereas all criterion variables except incomplete words primarily contributed to the synthetic criterion variable. As all of these structure coefficients had the same sign, results from the variate suggest that having higher attentional focusing and inhibitory control at age 54 months predicted better concurrent problem solving, letter-word identification, memory for sentences, and picture vocabulary and, in kindergarten, better teacher-rated math, and language and literacy. We labeled the dimension represented by the relationships in this variate “Effortful Control,” given its reflection of Rothbart and Bates’ [92] conceptualization of Effortful Control comprising attentional networks and inhibitory control. 

### 3.2. To What Extent Do Constellations of Temperamental Traits Predict the Social-Emotional Readiness of Kindergarten Students, as Indicated by the Nature of a Students’ Peer Interactions?

Canonical correlation analysis of eight temperament dimensions predicting five peer interactions variables yielded five canonical variates. The full model including all functions was significant (Wilks’s λ = 0.727; *F* [40, 2701.95] = 5.131, *p* < 0.001), where the full model explained 27.3% of the variance between the temperament variable set and the peer interactions variable set. Only the first and second variates were statistically significant; subsequent variates were not significant at *p* < 0.05. The first variate explained a considerable portion of shared variance between variable sets ($R_{s}^{2}$ = 0.182) and the second function also explained a considerable portion of the remaining variance ($R_{s}^{2}$ = 0.078), while subsequent functions (3 to 5) explained very small and non-significant portions of shared variance ($R_{s}^{2}$ = 0.025 to $R_{s}^{2}$ = 0.003). Standardized canonical function coefficients and structure coefficients for the first and second variates of the temperament and peer interactions variable sets are presented in Table 2. Examining the coefficients of Function 1, the predictor variables activity level, inhibitory control, and shyness comprised a considerable portion of the synthetic predictor variable, whereas the peer interactions outcome variables cooperation and self-control primarily contributed to the synthetic criterion variable. All structure coefficients had the same sign with the exception of activity level, suggesting that lower levels of inhibitory control and shyness and higher levels of activity were related to less cooperation and self-control. We labeled this variate “Dysregulated in Social Contexts.”

Examining coefficients for the second variate, predictor variables attentional focusing, inhibitory control, and shyness comprised a considerable portion of the synthetic predictor variable, whereas the outcome variables assertion and cooperation primarily contributed to the synthetic criterion variable. All structure coefficients had the same sign with the exception of shyness, suggesting that lower levels of attentional focusing and inhibitory control, but higher levels of shyness predicted less assertion and cooperation. We labeled this function “Withdrawn in Social Contexts.”

### 3.3. To What Extent Do Constellations of Temperamental Traits Predict the Social-Emotional Readiness of Incoming Kindergarten Students, as Indicated by Teacher-Child Relationship Quality?

Canonical correlation analysis of eight temperament dimensions predicting two teacher-child relationship variables yielded two variates. The full model including all functions was significant (Wilks’s λ = 0.885; *F* [16, 1282] = 5.025, *p* < 0.001), explaining 11.5% of the variance between the temperament variable set and the teacher-child relationship variable set. Examination of the dimension reduction analysis indicated that both canonical variates were statistically significant, with the first explaining a considerable portion of shared variance between variable sets ($R_{s}^{2}$ = 0.077), and the second also explaining a considerable portion of the remaining variance ($R_{s}^{2}$ = 0.041). Standardized canonical function coefficients and structure coefficients for the variates from the temperament and teacher-child relationship variable sets are presented in Table 3. Examining the coefficients of the first variate, the predictor variables activity level, inhibitory control, and shyness comprised a considerable portion of the synthetic predictor variable, whereas the outcome teacher-child conflict primarily contributed to the synthetic criterion variable. Teacher-child conflict and activity level had the same sign, while inhibitory control and shyness held the opposite sign, suggesting that higher activity level and lower levels of inhibitory control and shyness were related to more teacher-child conflict. We labeled this function “Highly Surgent.”

Examining coefficients for the second variate, a considerable portion of the synthetic predictor comprised predictor variables anger/frustration and inhibitory control, whereas both outcome variables contributed to the synthetic criterion variable. Although teacher-child closeness contributed more than teacher-child conflict ($r_{s}^{2}$ = 0.9197 versus $r_{s}^{2}$ = 0.2829, respectively). Closeness and inhibitory control had the same sign, which was opposite of the sign for conflict and anger/frustration, indicating that more inhibitory control and less anger/frustration were related to higher levels of closeness and lower levels of conflict. We labeled this function “Emotionally Controlled.”

## 4. Discussion

In this study, we used canonical correlation analysis to identify certain clusters of temperament traits that represent either protective or risk factors related to critical social and academic readiness indicators for children as they transition to formal schooling. The importance of school readiness in determining a child’s academic achievement trajectory has been well-established [93,94,95]. By highlighting the impact of temperament on these factors, we hope to provide meaningful insights for parents, teachers, and policy-makers who are tasked with preparing young children for a successful transition to this new stage of life.

These results suggest that certain temperamental traits are associated with the likelihood that a child will have a successful transition to school, especially in terms of academic and socio-emotional competencies. A key finding indicated that, for all desirable outcomes, the more children are able to regulate their behavior at 54 months, the more likely they are to be successful in Kindergarten in terms of attaining key academic skills and establishing positive relationships with teachers and successful interactions with peers. Here, regulation refers to effortful control, the complementary traits of attentional focusing and inhibitory control. These findings were not surprising, as the impact of a child’s regulatory abilities on school achievement has been well-documented (e.g., [4,9,96]).

In addition to these general findings, certain clusters of temperament traits were notable for their prediction of specific outcomes. First, in terms of attainment of academic skills in both pre-kindergarten (i.e., problem solving, letter word identification, memory for sentences, and picture vocabulary) and kindergarten (general math, language, and literacy growth), the two temperament dimensions indicative of effortful control (inhibitory control and attentional focusing) consistently emerged as predictors. Second, in terms of *social* readiness, effortful control, shyness, and activity level were consistent predictors of peer interactions. For example, results suggest that a child with higher levels of inhibitory control and shyness, and lower levels of activity at 54 months, is more likely to show cooperation and self-control (but, notably, not assertiveness) in kindergarten peer interactions. This finding was somewhat surprising based on research on shyness (cf. [66]), however, we surmised that inhibitory control may be exerting a protective effect on shyness as a trait. That is, the initial reaction to withdraw may be counterbalanced by the regulatory ability to engage positively with peers. In social contexts, this seems to describe the proverbial “quiet, unproblematic” child. However, this child also might be at risk in terms of maladaptation to peer contexts and lack of development of social competence due to shyness [44,97]. On the other hand, a child who is lower in shyness and inhibitory control, and higher in activity, may show signs of “hyperactivity” and, thus, struggle with cooperation and self-control during kindergarten peer interactions due to a difficulty with regulating externalizing behaviors [98].

Children’s levels of cooperation and assertiveness in peer interactions were predicted by a cluster of temperament traits that included high levels of attentional focusing and inhibitory control and lower levels of shyness. It is unsurprising that a child with these traits in preschool would be both more cooperative and more assertive in Kindergarten, as these traits describe a child who is both regulated and not withdrawn in peer interactions; these traits have been shown to work together in prior work predicting teacher-child relationship quality [99]. Conversely, a child who is lower in attentional focusing and inhibitory control and higher in shyness, may have more difficulty socially, as he or she may lack the “protective” benefits of high effortful control enjoyed by the child profiled in the first canonical variate. This child may avoid social interaction as a way of compensating for lower effortful control [100,101].

Similarly, inhibitory control, activity level, shyness, and anger/frustration emerged as predictors of teacher-child relationship quality in kindergarten. For conflict in the teacher-child relationship, a “highly surgent” child emerged (i.e., one who was higher in activity level, but also lower in both inhibitory control and shyness) who was likely to have more conflict with teachers, but not less closeness. This is congruent with research showing that conflict and closeness are not independent constructs, and that more boisterous children may get more teacher attention, both good and bad [59]. In contrast, we also found that children characterized as “emotionally controlled” (i.e., having higher inhibitory control and less anger and frustration) were more likely to have both lower conflict and higher closeness with their teachers in kindergarten. This finding was not surprising, given that high levels of anger have been associated with lower levels of self-regulation in children and adults [102]. It is also consistent with previous work showing difficulty in the teacher-child relationship for children with these temperament traits-termed “high maintenance” [103,104]. A student’s emotional volatility may impede the establishment of a positive relationship with the teacher, who may fall into a pattern of negative interactions with this type of student; this is concerning because higher conflict environments only serve to increase emotional reactivity and vigilance in anger-prone individuals [105,106].

Overall, these results highlight the importance of enhancing the regulatory capacities of preschool children, a conclusion that is validated with burgeoning evidence from studies with general and at-risk populations (e.g., [2,104,107,108]. As children move into their formal years of school, it appears to be particularly helpful for them bring along regulatory skills. The kindergarten classroom contains numerous demands for children’s behavioral regulation, such as sitting still, paying attention, following directions, taking turns, waiting in line, raising hands, keeping hands to self, working with others, and sharing supplies. Children who are able to successfully navigate the demands of the classroom will be get a bigger dose of academic and social experience, thus growing their skills in these interrelated areas [109].

This study also corroborates evidence that a child’s temperament can be a source of strength or a potential vulnerability as they transition to kindergarten. Parents’ and teachers’ interventions and interactions with children in the preschool years, and even earlier, would do well to encourage the development of focus, persistence, and emotional regulation, and to help children learn to regulate their emotions through conflict resolution. An example of a successful intervention for children in kindergarten and first grade is Insights into Children’s Temperament (INSIGHTS; [104]). INSIGHTS teaches children, teachers, and parents about temperament, and provides temperament-based strategies for managing conflict and behavior. Studies of the efficacy of INSIGHTS have demonstrated that children have better behavior, social skills, attention, and academic performance when they are in classrooms using the INSIGHTS program [104]. This is particularly true for children who have more demanding temperament profiles, such as high levels of anger/frustration and low levels of effortful control [103] and children who are high in shyness [110]. Future research into the development and effectiveness of strategies targeting the needs of children with more vulnerable or “high risk” temperament profiles is also warranted.

This study had several limitations. First, canonical correlation analyses software requires listwise deletion, a less-than-ideal method of handling missing data. Thus, each set of analyses had a different sample size depending on the number of individuals with complete data. However, our findings are consistent with the larger literature linking temperament and academic and social skills in young children in separate analyses, so we remain confident in our findings. Second, our sample is relatively homogenous in terms of race/ethnicity and socioeconomic status. Third, there are other variables that were not accounted for in our analyses that could have played a role, such as family income and classroom quality. Canonical correlation, although providing a multivariate approach to examining associations between clusters of variables, is not conducive to covariates. Although this study was exploratory in nature, it establishes the groundwork for future studies to explore interactions between multivariate temperamental constellations and covariates (e.g., gender or environmental factors) to inform the development of “goodness-of-fit” interventions which better adapt to the interplay of temperamental traits within individuals upon entering school.

## 5. Conclusions

The main findings of this study clarified the importance of temperamental self-regulation (effortful control) in predicting successful kindergarten transitions in the sample. Further, this study identified temperamental clusters associated with specific school readiness outcomes. Academic skills were specifically associated with effortful control (inhibitory control and attentional focusing). Social readiness was associated with a constellation comprising effortful control, shyness, and activity level. Children’s levels of cooperation and assertiveness in peer interactions were predicted by a temperamental cluster with high levels of attentional focusing and inhibitory control and lower levels of shyness. Conflict in the teacher-child relationship in kindergarten was associated with a constellation of high activity, but low inhibitory control and shyness (i.e., “highly surgent” constellation). A high conflict/low closeness relationship in kindergarten was related to the constellation comprising low inhibitory control and high anger and frustration (i.e., “emotionally controlled”).

## Figures and Tables

**Table 1:** Canonical solution for temperament predicting school readiness for variate 1.^1.^

Variable	V1 (Effortful Control)	
	Canonical	Structure
WJ-R Applied Problems	0.353	**0.840**
WJ-R Incomplete Words	−0.163	0.403
WJ-R Letter-Word ID	0.251	**0.767**
WJ-R Memory for Sentences	0.269	**0.647**
WJ-R Picture Vocab	0.158	**0.654**
ARS Lang & Literacy	0.347	**0.783**
ARS Math	0.039	**0.718**
Attentional Focusing	0.652	**0.873**
Activity Level	0.153	−0.375
Anger/Frustration	0.248	−0.103
Approach	−0.005	−0.114
Fear	−0.048	−0.089
Inhibitory Control	0.629	**0.791**
Sadness	0.024	−0.023
Shyness	0.118	0.092

^1^: Structure coefficients (rs) larger than ±0.45 are bolded. *Canonical* = standardized canonical function coefficient; *Structure* = structure coefficient. *V* = Variate.

**Table 2:** Canonical solution for temperament predicting peer interactions for variates 1 and 2.

Variable	V1 (Dysregulated)		V2 (Withdrawn)	
	Canonical	Structure	Canonical	Structure
SSRS Assertion	0.696	0.148	−0.921	**−0.833**
SSRS Cooperation	−0.938	**−0.817**	−0.578	**−0.522**
SSRS Self-Control	−0.132	**−0.492**	0.730	−0.119
Peer Status	−0.181	−0.378	−0.107	−0.433
Well Liked By Peers	0.012	−0.263	0.064	−0.446
Attentional Focusing	−0.111	−0.407	−0.444	**−0.615**
Activity Level	0.061	**0.544**	−0.389	0.043
Anger/Frustration	0.025	0.314	−0.019	0.198
Approach	−0.068	0.311	0.201	0.082
Fear	−0.050	−0.072	−0.213	−0.022
Inhibitory Control	−0.579	**−0.704**	−0.596	**−0.605**
Sadness	0.184	0.099	−0.208	0.029
Shyness	−0.721	**−0.701**	0.627	**0.592**

^1^: Structure coefficients (rs) larger than ±0.45 are bolded. *Canonical* = standardized canonical function coefficient; *Structure* = structure coefficient. *V* = Variate.

**Table 3:** Canonical solution for temperament predicting teacher-child relationship for variates 1 and 2. ^1.^

Variable	V1 (Highly Surgent)		V2 (Emotionally Controlled)	
	Canonical	Structure	Canonical	Structure
Teacher-child Closeness	0.552	0.283	0.880	**0.959**
Teacher-child Conflict	0.996	**0.847**	−0.294	**−0.532**
Attentional Focusing	0.135	−0.154	0.131	0.411
Activity Level	0.304	**0.538**	0.455	−0.141
Anger/Frustration	0.004	0.290	−0.558	**−0.514**
Approach	−0.176	0.280	−0.062	−0.121
Fear	−0.058	−0.064	0.244	0.174
Inhibitory Control	−0.391	**−0.510**	0.721	**0.705**
Sadness	0.437	0.235	0.543	0.197
Shyness	−0.790	**−0.759**	−0.277	−0.212

^1^: Structure coefficients (rs) larger than ±0.45 are bolded. *Canonical* = standardized canonical function coefficient; *Structure* = structure coefficient. *V* = Variate.

## Data Availability

Restrictions apply to the availability of these data. Data were obtained from the Inter-University Consortium for Political and Social Research (ICPSR) and are available at www.icpsr.umich.edu with the permission of ICPSR.
